# Renal Nitric Oxide Deficiency and Chronic Kidney Disease in Young Sheep Born with a Solitary Functioning Kidney

**DOI:** 10.1038/srep26777

**Published:** 2016-05-26

**Authors:** Reetu R. Singh, Lawrence K. Easton, Lindsea C. Booth, Markus P. Schlaich, Geoffrey A. Head, Karen M. Moritz, Kate M. Denton

**Affiliations:** 1 Cardiovascular Program, Monash Biomedicine Discovery Institute and Department of Physiology, Monash University, Melbourne, Australia; 2 The Florey Institute of Neuroscience and Mental Health, Melbourne Australia; 3 Baker IDI Heart and Diabetes Institute, Melbourne, Australia; 4 School of Biomedical Sciences, The University of Queensland, Brisbane, Australia

## Abstract

Previously, we demonstrated that renal hemodynamic responses to nitric oxide (NO) inhibition were attenuated in aged, hypertensive sheep born with a solitary functioning kidney (SFK). NO is an important regulator of renal function, particularly, in the postnatal period. We hypothesized that the onset of renal dysfunction and hypertension in individuals with a SFK is associated with NO deficiency early in life. In this study, renal and cardiovascular responses to L-NAME infusion (N^w^-nitro-L-arginine methyl ester) were examined in 6-month old lambs born with a SFK, induced by fetal unilateral nephrectomy (uni-x). Renal responses to L-NAME were attenuated in uni-x sheep with the fall in glomerular filtration rate (GFR) and urinary sodium excretion (U_Na_V) being less in the uni-x compared to sham lambs (%ΔGFR; −41 ± 3 vs −54 ± 4: P = 0.03, %ΔU_Na_V; −48 ± 5 vs −76 ± 3, P = 0.0008). 24 hour-basal urinary nitrate and nitrite (NOx) excretion was less in the uni-x animals compared to the sham (NOx excretion μM/min/kg; sham: 57 ± 7; uni-x: 38 ± 4, P = 0.02). L-NAME treatment reduced urinary NOx to undetectable levels in both groups. A reduction in NO bioavailability in early life may contribute to the initiation of glomerular and tubular dysfunction that promotes development and progression of hypertension in offspring with a congenital nephron deficit, including those with a SFK.

In children born with only one kidney (congenital solitary functioning kidney; SFK) or those who lose a kidney early in life (acquired SFK), the onset of hypertension and renal disease occur early in life^1^,^2^, and ~20–40% of these children develop end-stage renal disease (ESRD) by the age of 30^3^. In contrast, in adults who donate a kidney, the risk of developing chronic kidney disease (CKD) and hypertension are relatively low^4^ indicating that the loss of renal mass early in life may carry a greater risk for future onset of diseases but the mechanisms remain unclear. A relationship between small kidney length, renal dysfunction and arterial pressure in children with a SFK has been identified^1^. Additionally, infants born of low birth weight and those born premature, have smaller kidneys and in these children a higher prevalence of cardiovascular and CKD has also been reported^5^. Since a small kidney size correlates with low nephron number^6^, and given the importance of kidney function in regulation of arterial pressure, it is likely that alterations in factors regulating renal physiology early in life when the kidneys are undergoing functional maturation, underpin the development of hypertension in these children.

Nitric oxide is an important regulator of renal hemodynamics and tubular function^7^ and NO produced within the kidney contributes to the regulation of sodium excretion and thus, maintenance of vascular volume and arterial pressure in the adult^8^. NO also plays a significant role in the normal maturation of renal function early in the postnatal period. Renal blood flow (RBF) and GFR are low in the fetus but increase rapidly after birth^4^. In newborn lambs, the rise in RBF during the postnatal period occurs with a concomitant increase in nitric oxide (NO) production^9^. Moreover expression of endothelial NO synthase (eNOS) increases progressively in the pre-glomerular resistance vasculature of the newborn compared to the adult suggesting a critical role for NO in modulating renal hemodynamics in the postnatal period^10^. A reduction in bioavailability/production of NO has been observed in experimental models of nephron deficiency^11^,^12^ and in patients with ESRD and hypertension^13^. Additionally it has been demonstrated that increasing NO bioavailability by L-Arginine or Citrulline supplementation normalizes blood pressure, improves renal function and prevents proteinuria in developmental programming^14^ and genetic models^15^ of hypertension. This suggests that a deficiency of NO is present in both genetic and acquired forms of hypertension. Therefore, a strong case can be made for NO deficiency in the early life as a primary stimulus for the development and/or progression of hypertension and renal disease in adulthood^16^,^4^,^17^.

Since majority (~90%) of children with SFK do not have extra-renal abnormalities^18^, we established an ovine model of congenital SFK to better understand the effects of a reduction of renal mass on regulation of renal and cardiovascular function. In our model, a congenital SFK is induced by performing unilateral nephrectomy in the sheep fetus (uni-x) at 100 days of gestation (term = 150 days) and this results in ~30% reduction in total nephron number as a result of some compensatory nephrogenesis in the remaining kidney of the sheep fetus^19^. The sheep begins formation of the permanent kidney at day 27 of gestation and completes nephrogenesis at day 130 of gestation, 3 weeks prior to birth^20^, making it almost identical to the human which also completes nephrogenesis 3 weeks prior to birth^20^. Similar to our observations in sheep, compensatory nephrogenesis has also been reported in children with SFK^21^. We have demonstrated that both male and female uni-x sheep have early onset of disease with increases in arterial pressure and reductions in glomerular filtration rate (GFR) occurring by 6 months of age^22^,^23^. Additionally, the normal age-related decline in renal function and elevation in blood pressure is exacerbated by 4–5 years of age^24^,^25^. Our detailed characterization of renal function in aged sheep with SFK demonstrated that renal hemodynamic responses to inhibition of nitric oxide synthase (NOS) were attenuated^26^ indicating a nitric oxide (NO) deficiency. However, a limitation of the study in aged sheep was that the hypertension was longstanding (~4 years) and therefore it was not possible to determine if the NO deficiency was a cause or a consequence of the hypertension. Studies in rodents have also implicated reduced NO bioavailability in the developmental origins of hypertension, though again these studies were conducted once hypertension was well established^27^.

In the present study we examined if a deficiency of NO was present in young sheep with a congenital SFK (induced by fetal uni-x). Our studies in the young sheep are important because very little is known about the mechanisms driving the early onset of disease in children with SFK. We hypothesized that a renal NO deficiency is present early in life in lambs with a congenital SFK (uni-x) such that the contribution of NO to basal renal hemodynamics and function would be reduced. Specifically in the present study cardiovascular and renal responses to *in vivo* NOS inhibition via the administration N^w^-nitro-L-arginine methyl ester (L-NAME) in conscious female lambs with a SFK or with intact kidneys was examined at 6 months of age. Additionally we examined levels of urinary nitrate as a marker of NO deficiency^22^.

The findings of the current study are novel because for the first time, we have identified a change in a factor regulating renal hemodynamic very early in life that provides an early biomarker of disease. The finding that our sheep with a congenital SFK have deficiencies in NO from as early as 6 months of age, a time when they are only very mildly hypertensive suggests this mechanism contributes to the overt and progressive elevations in blood pressure that occur with aging^25^. Having identified a deficiency of NO early in the disease phase, future studies can examine whether increasing NO bioavailability from this stage can be a viable treatment option for slowing the progression of disease in those with congenital SFK.

## Results

### Body weights

All lambs were born at 150 ± 1 days of gestation. Body weight at birth and at 6 months of age, was not different between the sham and uni-x groups (birth weight (kg): sham: 3.5 ± 0.3, uni-x: 4.2 ± 0.2; 6 months (kg): sham: 24.5 ± 1.7, uni-x: 25.5 ± 0.8).

### Metabolic cage-balance studies

**Figure 1:**
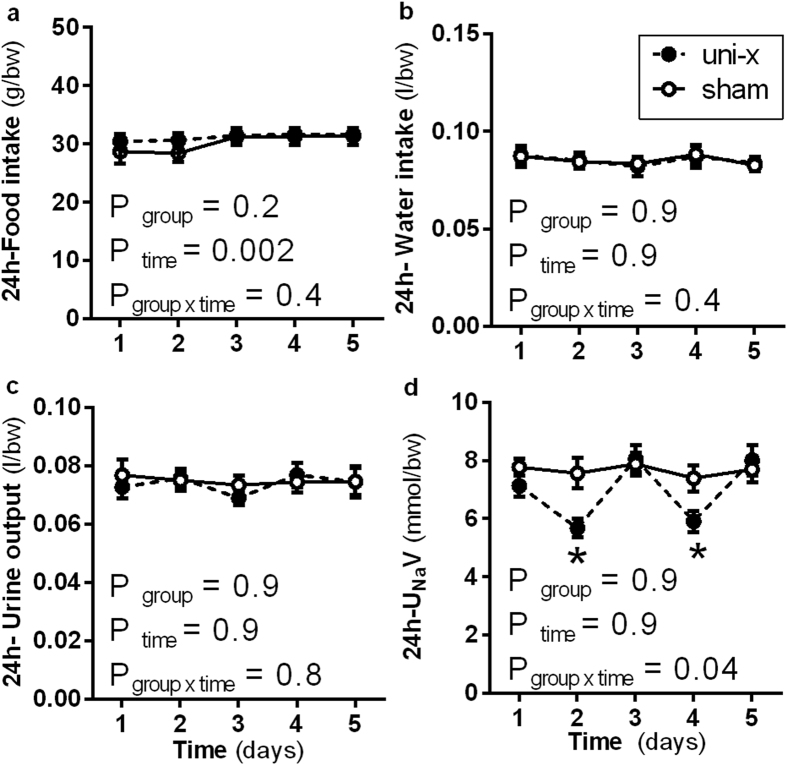
Twenty-four hour (**a**) food intake, (**b**) water intake and (**c**) urine output and urinary sodium excretion (U_Na_V) measured over a period of 5 days at 6 months of age in sheep that underwent either uninephrectomy (uni-x; n = 9; solid circles) or sham (n = 7; open circles) surgery on 100 days of gestation. P-values are from a repeated measures ANOVA with factors group (sham, uni-x), time and their interaction. *P < 0.05 Bonferroni post- hoc as compared to the sham group. Variables are expressed per bodyweight (bw). Values are presented as mean ± sem.

Food intake, water intake, urine output and urinary sodium excretion (U_Na_V) during the 5 days the animals were housed in metabolic cages are shown in Fig. 1. All variables were corrected for body weight. Food intake increased in both groups to a similar extent across time (P_time_ = 0.002; P_group x time_ = 0.4, Fig. 1a). Both groups had similar 24-h water intake (Fig. 1b) and urine output (Fig. 1c) over the 5-day period. In the sham lambs U_Na_V was similar across each day of the measurement period. However, in the uni-x lambs U_Na_V oscillated, increasing and decreasing across the 5-day period, and this was significantly different to the response in the sham group (P_group x time_ = 0.04, Fig. 1d). However, average U_Na_V across the 5-day period was not significantly different (average U_Na_V, mmol/min/bw; sham: 7.6 ± 0.1, uni-x: 7.0 ± 0.5, P = 0.2).

### Basal 72-h arterial pressure and heart rate

**Figure 2:**
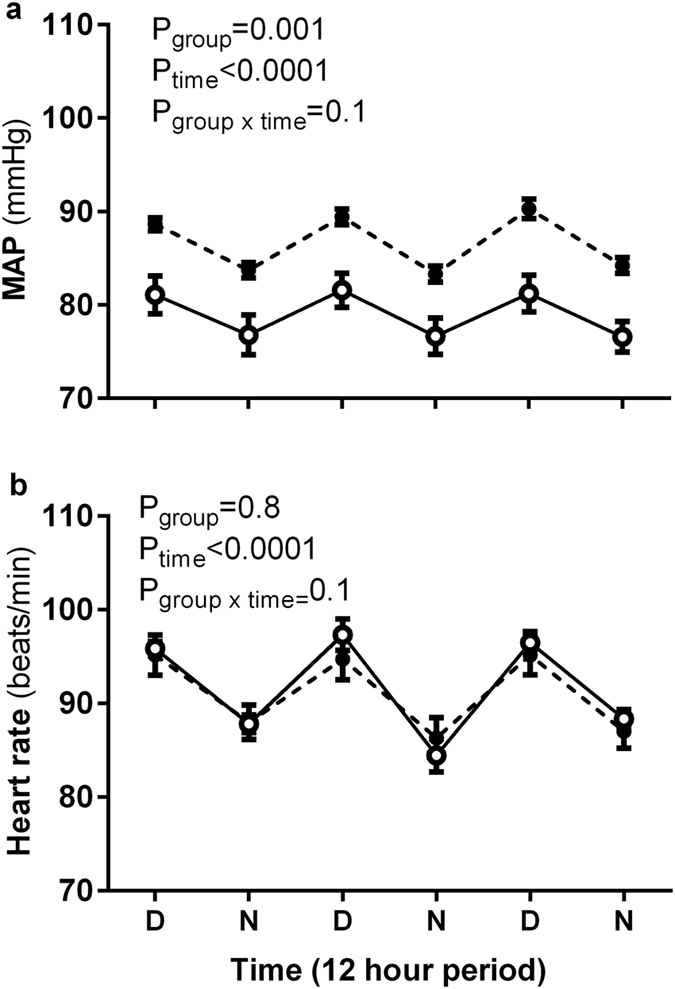
Continuous three day measurement of (**a**) mean arterial pressure (MAP) and (**b**) heart rate (HR) presented as day and night averages at 6 months of age in un-anesthetised sheep that underwent either uninephrectomy (uni-x; n = 9; black bars) or sham (n = 7; open bars) surgery at 100 days of gestation. P-values are from a repeated measures ANOVA with factors group (sham, uni-x), time and their interaction. Values are presented as mean ± sem.

Mean arterial pressure (MAP) and heart rate (HR), continuously recorded in un-anesthetized animals over 72 hours, are reported as averages of the day (5 am–5 pm) and night period (5 pm–5 am) (Fig. 2). The diurnal rhythm was similar in both groups for MAP (P_time_ < 0.0001, P_group x time_ = 0.1, Fig. 2a) and HR (P_time_ < 0.0001, P_group x time_ = 0.1, Fig. 2b). Uni-x animals had a significantly higher MAP compared to the sham group (average 72-h: MAP (mmHg); sham: 80 ± 2, uni-x: 86 ± 1, P = 0.009, Fig. 2a). but HR was similar between the groups (average 72-h: Heart rate (beats/min); sham: 91.7 ± 2.2, uni-x: 91.1 ± 1.8, Fig. 2b).

### Basal cardiovascular and renal response to vehicle infusion

**Figure 3:**
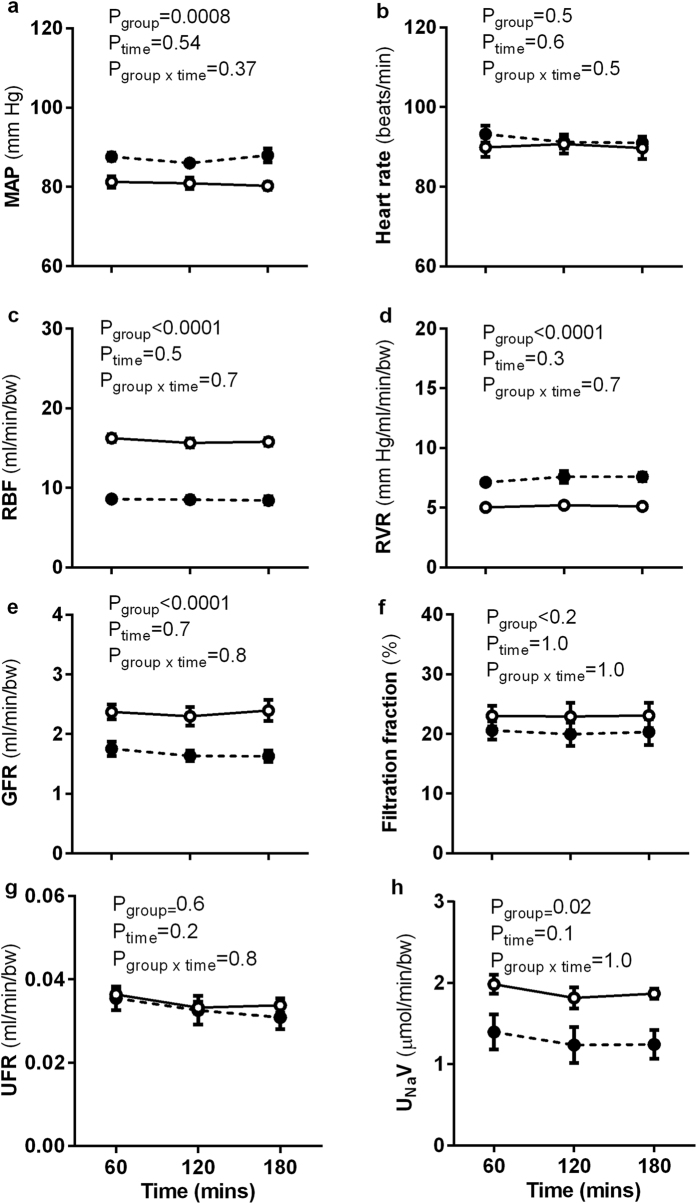
Cardiovascular and renal responses to vehicle infusion. **(a**) Mean arterial pressure (MAP), (**b**) heart rate (HR), (**c**) renal blood flow (RBF), (**d**) renal vascular resistance (RVR), (**e**) glomerular filtration rate (GFR), (**f**) filtration fraction, (**g**) urine flow rate (UFR), (**h**) urinary sodium excretion (U_Na_V) measured over 3 hours of vehicle infusion in sheep aged 6 months that underwent fetal uninephrectomy (uni-x; n = 9; closed symbols-broken lines) or sham surgery (n = 7; open symbols-solid lines). P-values are from a repeated measures ANOVA with factors group (sham, uni-x) and time (period of vehicle infusion) and their interaction (group x time). Renal variables are expressed per bodyweight (bw). All values are presented as mean ± sem.

Cardiovascular and renal function were also assessed in response to a 3 hour period of vehicle infusion as a time control (Fig. 3). MAP was significantly higher in uni-x animals compared to sham animals (P_group_ = 0.0008, Fig. 3a) and heart rate was not different between the groups (Fig. 3b). No effect of time was observed on either of these variables.

RBF, renal vascular resistance (RVR), GFR and filtration fraction did not vary with time in either treatment group (Fig. 3c–f). Uni-x animals had a significantly lower RBF (P_group_ < 0.0001, Fig. 3c) and GFR (P_group_ < 0.0001, Fig. 3e) compared to the sham group across the entire 3 hour period. RVR was greater in uni-x animals compared to the sham group across the entire 3 hour period (P_group_ < 0.0001, Fig. 3d) but filtration fraction was similar between the groups (Fig. 3f). Urine flow rate (UFR) was similar between the groups across the 3 hour period of measurement and was not affected by time (Fig. 3g). Uni-x animals had a lower urinary excretion of sodium (U_Na_V) compared to the sham (P_group_ = 0.02, Fig. 3h) but time had no effect on U_Na_V in either treatment group.

### Cardiovascular and function in response to L-NAME infusion

**Figure 4:**
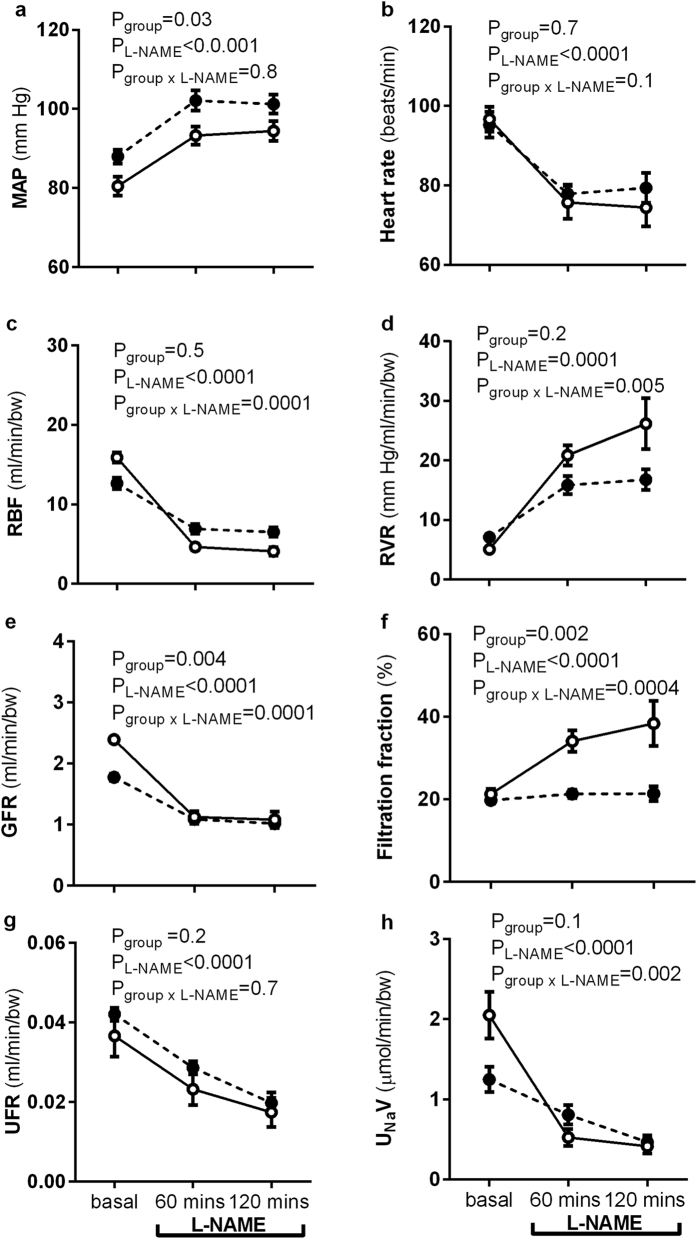
Cardiovascular and renal responses to L-NAME infusion. (**a**) Mean arterial pressure (MAP), (**b**) heart rate (HR), (**c**) renal blood flow (RBF), (**d**) renal vascular resistance (RVR), (**e**) glomerular filtration rate (GFR), (**f**) filtration fraction, (**g**) urine flow rate (UFR), (**h**) urinary sodium excretion (U_Na_V) measured over 1 hour (basal) and in response to 120 minutes of infusion of L-NAME in sheep aged 6 months that underwent fetal uninephrectomy (uni-x; n = 9; closed symbols-broken lines) or sham surgery (n = 7; open symbols-solid lines). P-values are from a repeated measures ANOVA with factors group (sham, uni-x) and treatment (before and during L-NAME) and their interaction (group x treat). Renal variables are expressed per bodyweight (bw). All values are presented as mean ± sem.

Prior to administration of L-NAME cardiovascular and renal function was assessed over a period of 1 hour to establish a baseline on that day (Fig. 4). During the hour baseline period the variables measured were similar to those obtained during the assessment of renal function during vehicle infusion (Fig. 3).

The responses to L-NAME infusion were similar over the 2 hours of administration (Fig. 4), therefore this data was averaged to allow determination of the average response to L-NAME across the infusion period to compare the percent response in each group. In response to L-NAME, MAP increased compared to baseline in both groups (both P < 0.0001, Fig. 4a). The percentage change in MAP in response to L-NAME from baseline was similar between the groups (%Δ MAP; sham: 17 ± 1, uni-x: 16 ± 2). HR decreased in response to L-NAME in both groups compared to baseline (both P < 0.0001, Fig. 4b). The percentage change in HR from baseline in response to L-NAME was not different between the groups (%Δ HR; sham: −23 ± 3, uni-x: −18 ± 1).

In response to L-NAME, GFR and RBF decreased compared to baseline in both sham and uni-x groups (both P < 0.0001, Fig. 4c,e). However, the fall in GFR (%Δ GFR; sham: −54 ± 4, uni-x: −41 ± 3, P = 0.03) and RBF (%Δ RBF; sham: −73 ± 3, uni-x: −47 ± 3, P < 0.0001) were less in the uni-x compared to sham lambs. In response to L-NAME, RVR increased in both groups compared to baseline (both P < 0.001, Fig. 4c). However, the percent change in RVR from baseline in response to L-NAME was significantly less in the uni-x group relative to the sham (%Δ RVR; sham: 349 ± 44, uni-x: 125 ± 12, P < 0.0001). As a result of the proportionally larger fall in RBF as compared to GFR in the sham group, filtration fraction was observed to increase in response to L-NAME (P < 0.0001, Fig. 4f). However, as GFR and RBF fell by a similar extent in response to L-NAME in the uni-x lambs, filtration fraction was not different to baseline (Fig. 4f).

UFR decreased in response to L-NAME compared to baseline in both groups (both P < 0.001) but the percentage change was not different between the groups (%Δ UFR; sham: −50 ± 7, uni-x: −42 ± 5, Fig. 4g). U_Na_V declined in response to L-NAME compared to baseline in both groups (sham, P < 0.0001, uni-x, P < 0.01, Fig. 4h). Moreover, the percent change in U_Na_V in response to L-NAME from baseline was less in the uni-x compared to sham lambs (%ΔU_Na_V; sham: −76 ± 3, uni-x: −48 ± 5, P = 0.0008).

### Urinary NOx

Basal 24-h urinary nitrate and nitrite (total NOx) excretion was significantly less in the uni-x compared to the sham animals (total NOx excretion; μM/min/bw; sham: 57 ± 7; uni-x: 38 ± 4, P = 0.02). Similarly during the hour baseline period on the day of the L-NAME infusion NOx excretion was lower in the uni-x as compared to the sham lambs (NOx excretion; μM/min/bw; sham: 72 ± 8; uni-x: 54 ± 3, P = 0.04). NOx levels could not be detected in urine at 120 mins of L-NAME administration (below the detection limit of the assay kit).

## Discussion

The major finding of this study was that NO made a reduced contribution to the modulation of renal hemodynamics and tubular function in young sheep born with a single kidney. This is the first study to demonstrate this in a large animal model of congenital solitary functioning kidney. Inhibition of NO synthases caused RBF and GFR to decrease in both the uni-x and sham lambs at 6 months of age, but the reduction was markedly less in the uni-x sheep. Furthermore, whilst filtration fraction almost doubled in the normotensive sham group, filtration fraction was unaltered in the uni-x animals. Additionally, total urinary NOx (nitrate and nitrite) excretion was significantly less in uni-x lambs indicative of reduced renal NO production. This data demonstrates NO deficiency is present in juvenile sheep subject to the removal of a kidney as a fetus and that the NO deficit has impacted control of net glomerular filtration pressure as evidenced by the lack of modulation of filtration fraction by L-NAME in the uni-x lambs. Thus, a reduction in NO bioavailability early in life may contribute to the chronic kidney disease and hypertension observed in children with a SFK.

Previously, in aged sheep (5 years old) we identified a NO deficit in the kidney of uni-x sheep^26^. A major limitation of that study was that the sheep had been hypertensive for 4 years at the time of study at 5 years of age. Thus, we could not determine if the NO deficiency was a cause or an effect of the hypertension. In the current study we demonstrated a diminished role for NO in the modulation of renal function in juvenile uni-x sheep (50% of their adult body weight at this age). In our study in aged animals, notably there was an aged related decline in the contribution of NO to the control of renal function, which was exacerbated in the uni-x sheep^26^. However, this renal NO deficit is still very much evident in the 6-month old uni-x lambs. In terms of basal renal function, we have shown that renal blood flow declined in the uni-x sheep significantly between the age of 1 year to 5 years.^25^ Given the important role of NO in maintaining renal hemodynamics, such a decrease in renal function is consistent with a decline in NO levels with ageing. However, renal NO has been shown to be a key modulator of increases in renal function which occur in the postnatal period^28^. Therefore, if a reduction in NO is present from a young age, this may limit the appropriate increases in renal function in the postnatal period and hasten the “normal” age-related decline in renal function. Our findings show that a reduction in NO production/bioavailabilty is present from early in life in the presence of a nephron deficit and this may be responsible for the greater decline in renal function and exacerbation of hypertension with age observed in sheep with a congenital SFK.

NO is a potent vasodilator and an important regulator of both glomerular and tubular function^7^. In response to systemic administration of L-NAME, in the normotensive sham lambs we observed an increase in MAP that was associated with an increase in RVR and falls in RBF, GFR, urine flow and sodium excretion similar to previous reports in sheep^29^ and other species^30^,^31^. MAP increased similarly in both the sham and the uni-x animals (~13 mmHg). This finding is comparable to our observations in aged female sheep in which a similar elevation in MAP (~17 mmHg) was observed in response to L-NAME in both sham and uni-x groups^26^. Alterations in systemic arterial pressure can, via autoregulation influence renal hemodynamics but given that the increase in arterial pressure was of a similar degree, it likely equally affected renal function in both groups. RVR comprises resistance in the pre- and post-glomerular segments of the renal vasculature. In young sham sheep, the increase in RVR in response to L-NAME was large (~300%) and was accompanied by unequal falls in GFR (54%) and RBF (72%) resulting in an increase in filtration fraction. Previous studies have also reported a lesser reduction in GFR than RBF following NOS inhibition, leading to an increase in filtration fraction^32^,^16^. This suggests that nitric oxide is acting predominantly at the efferent arterioles^33^. Collectively, these findings highlight the importance of endogenous NO within the kidney in the regulation of renal hemodynamics.

In marked contrast to the normotensive sham group, in the uni-x lambs the increase in RVR (~120%) and the decreases in GFR (41%) and RBF (47%) to NOS inhibition were attenuated. Since the decrease in GFR and RBF were of the same order, filtration fraction did not significantly change in uni-x animals in response to L-NAME, suggesting a similar degree of vasoconstriction at the afferent and efferent arterioles. NO has also be shown to raise GFR by increasing the glomerular ultrafiltration coefficient^33^ and NO deficiency might influence GFR by this mechanism but this was not addressed in the current study. Thus the kidney of uni-x sheep, due to the inability to recruit NO, may have an impaired ability to regulate net glomerular filtration pressure, and thus GFR, in response to changes in perfusion pressure. In the kidney, NO is produced by endothelial NOS (eNOS) in the vasculature and neuronal NOS (nNOS) in the macula densa cells, which acts to modulate segmental renal vascular resistance and glomerular dynamics^34^,^10^. Additionally, whilst urine flow decreased by a similar extent in both groups, the reduction in sodium excretion was attenuated in the uni-x (48%) as compared to the sham lambs (76%) in response to L-NAME. This observation is similar to our observations in aged sheep^26^. Under normal homeostatic conditions, NO has been shown to block sodium transporters and decrease sodium and fluid reabsorption^35^,^36^,^37^. Thus, our findings suggest that uni-x lambs likely have a NO deficiency in both the vascular and tubular components of the nephron.

NO deficiency associated with a reduction in renal mass and/or nephron number in early life may have far reaching effects. NO is involved in the maturation of renal function in the postnatal period^17^,^10^,^4^. RBF and GFR are low in the fetus but increase rapidly in the postnatal period^38^. In newborn lambs, the rise in RBF during the postnatal period^39^ occurs with a concomitant increase in NO production^9^. Moreover the ontogeny of expression of NOS in the glomerular resistance vasculature of the newborn compared to the adult suggests a critical role for NO in modulating renal hemodynamics in the postnatal period. In the renal resistance vasculature of the newborn pig, the expression of the neuronal isoform of NOS (nNOS) is greatest, but in the adult, eNOS has the greatest expression^10^. In support of this, nNOS makes a greater contribution to the control of RBF in the postnatal period but in the adult pig, eNOS contributes predominantly^10^. In the sheep kidney, all three isoforms of NOS have the greatest expression at birth, particularly in the renal cortex and the levels start to decline from 12 weeks of age^34^, providing strong evidence that NO plays an important role in the regulation of renal function in the early postnatal period^17^. Thus, NO deficiency may drive the development of hypertension and renal dysfunction when renal mass is reduced early in life, as a result of being born with a SFK or low nephron number.

Total urinary NOx excretion was significantly lower in the uni-x lambs, indicative of a reduction in endogenous NO synthesis. Previously, in a pediatric population lower urinary NOx has been associated with acute kidney injury^40^. Thus, urinary nitrate may be a predictive marker of future cardiovascular and renal risk in children with a SFK. The lambs in the present study are part of a larger on-going project and as such tissues were not collected and therefore it was not possible to further directly examine the mechanistic pathways causing NO deficiency in the uni-x lambs. However, experimental and clinical studies have demonstrated that renal dysfunction is associated with a state of low NO production^12^,^41^,^13^ and can be a result of increased levels of assymetric dimethylarginine (ADMA)^42^,^43^, a potent competitive inhibitor of all three isoforms of NOS or a reduction in NOS activity and/or NOS expression^12^,^44^. A reduction in NO bioavailability can also be brought about in the presence of increased NOS expression. It has been shown that under pathological conditions such as hypertension, an increase in eNOS expression is observed but there is also a reduction in eNOS dimer formation and eNOS uncoupling^45^,^46^. eNOS uncoupling is the enzymatic reduction of molecular oxygen coupled to L-arginine, resulting in the generation of the superoxide, rather than NO^47^ and eNOS dimerization is a mechanism for preventing eNOS uncoupling^48^. The increased generation of reactive oxygen species (ROS) can also in turn inactivate NO, for example the nitrosylation of NO^7^. In developmental programming models of hypertension, oxidative stress has been implicated in reducing NO bioavailability where nitric oxide is nitrosylated to generate peroxynitrites, which mediate oxidative injury^49^. Indeed in our aged uni-x sheep, in addition to attenuated renal responses to systemic administration of L-NAME, we showed that inhibition of eNOS in isolated renal lobar arteries resulted in significantly lower basal tone generation^26^. This suggests that NO generation is impaired under basal conditions. However, this response was observed in the presence of increased renal eNOS gene and protein expression in the aged uni-x animals^26^. Further investigation revealed that renal arteries from aged uni-x sheep had greater levels of 3-nitrotyrosine^26^ suggestive of increased ROS generation and oxidative stress. Oxidative stress has been suggested to be both a cause and consequence of renal injury and hypertension^50^. Whether, the mechanisms of reduction in production/bioavailability of NO in younger uni-x sheep is similar to that of the aged sheep warrants further investigation. Finally, recent studies have suggested that dietary nitrate has blood pressure lowering effects and that the renal vasculature is particularly sensitive to dietary nitrate, at levels at which other beds are unresponsive^51^. Thus, dietary nitrate supplementation may be a viable novel therapy for children with a SFK to limit the progression of renal injury and hypertension.

Finally, at 6 months of age female uni-x lambs had a higher MAP (~6–7 mmHg) and a lower GFR (~30%) compared to the sham lambs at 6 months of age in agreement with previous studies in this model^22^. These findings of hypertension and renal insufficiency are also consistent with reports in other models of nephron deficiency^52^,^53^. In addition, whilst sodium excretion varied less than 5% each day in the sham group, in the uni-x lambs it varied much more widely. In the female uni-x lambs daily U_Na_V fluctuated by as much as 25% each day despite food intake being similar to the control group. However, over the 5 day period the total sodium excretion was not different between the groups, rather the excretion level oscillated around the mean more widely in the uni-x group. This pattern of greater variation in daily sodium excretion has also been observed in male uni-x lambs aged 6 months^54^. Furthermore, it has been demonstrated previously that following a saline load uni-x sheep excrete a greater amount of sodium but at a slower rate than sham animals^54^,^55^. In similar studies in rats that underwent uninephrectomy early in life, a reduced ability to excrete a sodium load has also been observed^56^. This situation is in marked contrast to the events that occur following uninephrectomy in adults, which in a healthy adult has little impact on renal function or blood pressure^57^. This study provides convincing evidence. Together this data suggests that the impairment of sodium handling in young sheep following fetal uni-x is associated with a diminished role for NO in the modulation of renal function and this may underpin the development of hypertension following loss of a kidney in early life^4^,^58^.

## Conclusion

In conclusion, lambs born with a SFK have a renal NO deficit as demonstrated by an attenuated renal response to NOS inhibition and a lower total urinary NOx excretion. Therefore, alterations in the NO system could be an important prognostic marker for disease in the setting of a congenital nephron deficit. In addition, identification of the causes of the reduction in NO bioavailability warrants further investigation in this model. This could provide the groundwork for the NO system to be targeted early in life for individuals born with a SFK and individuals born with a suspected nephron deficit such as premature infants^59^ and infants that experience poor conditions in-utero^53^.

## Materials and Methods

All experiments were approved by an Animal Ethics Committee of Monash University and were performed in accordance with the guidelines of the National Health and Medical Research Council of Australia. Pure-bred merino ewes were time-mated and on gestational day 90, the pregnant ewes were transported to Monash University Large Animal Facility where they were housed in lambing pens and allowed at least 10 days to acclimatize prior to undergoing surgery, as previously described^60^. In brief, in the ewes, on gestation day 100, anesthesia was induced with intravenous administration of thiopentone sodium (20 mg/kg, Thiobarb, Lyppard Ltd, Australia) and maintained with isoflurane (1.5–2% in oxygen, Troy Laboratories Pty Ltd, Australia) for the duration of the surgery. After preparing the ewes for surgery, a midline incision was made, the uterus was manipulated and an incision was made to pull the hind-quarters of the fetal lamb out of the uterus. An incision was made over the left flank of the fetus and the left kidney was exposed and cleared of surrounding fat. Then, unilateral nephrectomy was performed by ligating the left renal artery, vein and ureter and excising the kidney (uni-x; n = 9). In 7 fetuses, the left kidney was exposed and gently manipulated but not excised (sham; n = 7). After recovery from surgery the ewes were returned to the farm and allowed to lamb naturally. Birth weight was recorded for all lambs. Lambs remained with their mothers on pasture until 18 weeks of age when they were weaned. At 4 months of age animals underwent surgery to construct carotid arterial loops. Anesthesia was induced as described above and the right and left carotid arteries were exteriorized into skin folds to enable direct access to the carotid artery for cannulations^22^. At the same time, the ewes underwent bilateral ovariectomy to minimize the effect of the oestrus cycle on blood pressure and renal function^22^. Each of the following four experiments, on separate days, were performed in un-anesthetized lambs.

### Metabolic cage-balance studies

At 6 months of age, lambs were brought into the animal housing facility and housed in pens for a week. Following this period of acclimation animals were weighed and housed individually in metabolic cages. These cages allow for the separation of feces from urine. Then, for a period of 5-days animals were meal-fed 800 g of lucerne hay and chaff and offered 5000 ml of water at 0900 h daily. Food intake, water intake and urine output over 24 hours were measured daily. A urine sample was collected each day to measure urinary sodium excretion. Additionally on the final day (day 5), urine was also collected to determine urinary nitrate concentration.

### 24-hour arterial pressure and heart rate

At 6 months of age 24-hour arterial pressure (mean, systolic and diastolic) and heart rate, over 3 days, were measured via a catheter (PVC tubing, 1.5 × 1.0 mm, Microtube Extrusions, NSW, Australia) inserted into the carotid artery. Following cannulation, the carotid arterial catheter was connected to a pressure transducer (pvb DPT-6000, Cellplex Pty Ltd, Australia) and a blood pressure acquisition unit. Measurements of blood pressure and heart rate were acquired in un-anesthetized lambs using the HEM Cardiovascular Monitoring system (Notochord Systems, Notochord, Japan) in real-time and averaged over 10 seconds.

### Cardiovascular and renal response to vehicle infusion

Basal renal function (time control experiment) was determined over a period of 3 hours during vehicle infusion (12 ml/h 0.9% isotonic saline, Baxter Healthcare, USA). A day prior to this, all animals had a catheter inserted into their right and left jugular vein (PE tubing, 1.2 × 1.0, Microtube Extrusions, Australia) for infusion purposes. On the morning of the experiment, a Foley catheter (Size 8 or 10, Uromedical, Australia) was inserted into the bladder for continuous urine collection. ^51^Chromium ethylenediaminetetraacetic acid (^51^Cr EDTA) and para-aminohippuric acid (PAH), was administered (i.v.) for the determination of glomerular filtration rate (GFR) and renal blood flow (RBF), respectively, via clearance methods as previously described^55^. An equilibration period of one hour was allowed for the ^51^Cr EDTA and PAH to reach steady-state within the plasma. Following the equilibration period, urine samples were collected at 20 minute intervals, with an arterial blood sample (5 ml) collected mid-point, for a period of 3 hours. Blood pressure and heart rate were monitored continuously during this period.

### Cardiovascular and function in response to L-NAME infusion

The renal function protocol above was repeated 2 days later to examine the effect of L-NAME administration. Cardiovascular and renal function were measured for a period of 1 hour to establish a baseline following which animals received L-NAME (40 mg/kg bolus + 20 mg/kg/h infusion, i.v., L-NAME hydrochloride, Cayman Chemical, Australia) for 2 hours. This dose of L-NAME has previously been shown to completely abolish responses to NO stimulation in conscious lambs^29^. Urine samples were collected every 20 minutes with a 5 ml arterial blood sample collected at each mid-point for the duration of the experiment. Urinary nitrate levels were determined before and at the end of L-NAME infusion (see below).

### Sample analysis

Urine and plasma samples were frozen and stored at −20 °C. ^51^Cr EDTA levels were measured using a gamma counter (PerkinElmer Wizard 1470). PAH concentration was determined using a using a previously described rapid microplate assay method^61^ and urinary sodium concentration was measured using an electrolyte analyser (RapidChem 744, EBOS). All renal variables were corrected for body weight.

Total urinary nitrate and nitrite concentration (NOx) was measured using a colorimetric assay (Cayman Chemical Company). NOx levels were determined in urine collected during the last day of the metabolic-cage study, and before and after 120 mins of L-NAME infusion. Samples were diluted in assay buffer (1:20) and assayed in duplicates as per the manufacturer’s instructions. Detection limit of the assay for NOx was 2.5 μM. The intra-assay coefficient of variation was 2.2%.

### Statistical analysis

Values are presented as mean ± sem. Statistical analysis was performed using Graphpad software (Graphpad Prism 6 for Windows, Graphpad Software Inc, USA), with the level of significance set at P≤0.05. The Kolmogorov-Smirnov test was used to determine if data fitted a Gaussian distribution before further statistical analysis. An unpaired, two-tailed student’s t-test was used to compare body weight and urinary NOx levels between the sham and uni-x groups. For the 5-day metabolic-cage balance and 24-h arterial pressure and heart rate data a repeated measures analysis of variance (ANOVA) was performed with factors group (P_group_; sham or uni-x), time (P_time_) and their interaction (P_group x time_). For the cardiovascular and renal variables during vehicle or L-NAME infusion, a repeated measures ANOVA was performed with factors group (P_group_; sham or uni-x), treat (P_treat;_ before and during treatment) and their interaction (P_group x treat_). A Bonferroni post-hoc test was performed to compare the responses to baseline or between groups as appropriate.

## Additional Information

**How to cite this article**: Singh, R. R. *et al*. Renal Nitric Oxide Deficiency and Chronic Kidney Disease in Young Sheep Born with a Solitary Functioning Kidney. *Sci. Rep.* **6**, 26777; doi: 10.1038/srep26777 (2016).
